# Current status and progress of concurrent chemoradiotherapy in patients with locally advanced non‐small cell lung cancer prior to the approval of durvalumab

**DOI:** 10.1111/1759-7714.13357

**Published:** 2020-02-14

**Authors:** Tomoya Fukui, Shinji Hosotani, Itaru Soda, Takahiro Ozawa, Seiichiro Kusuhara, Mikiko I. Kakegawa, Masashi Kasajima, Yasuhiro Hiyoshi, Satoshi Igawa, Masanori Yokoba, Hisashi Mitsufuji, Masaru Kubota, Masato Katagiri, Jiichiro Sasaki, Hiromichi Ishiyama, Katsuhiko Naoki

**Affiliations:** ^1^ Department of Respiratory Medicine Kitasato University School of Medicine Kanagawa Japan; ^2^ Department of Radiology and Radiation Oncology Kitasato University School of Medicine Kanagawa Japan; ^3^ Department of Medical Laboratory Kitasato University School of Allied Health Sciences Kanagawa Japan; ^4^ Fundamental Nursing Kitasato University School of Nursing Kanagawa Japan; ^5^ Research and Development Center for New Medical Frontiers Kitasato University School of Medicine Kanagawa Japan

**Keywords:** Chemoradiotherapy, immune checkpoint inhibitor, locally advanced, non‐small cell lung cancer, radiation pneumonitis

## Abstract

**Background:**

The standard treatment for patients with unresectable locally advanced (LA) non‐small cell lung cancer (NSCLC) is chemoradiotherapy (CRT). Consolidation therapy with durvalumab after CRT demonstrated survival benefits and was approved in Japan in July 2018. The use of immune checkpoint inhibitors (ICIs) is entering routine oncological practice, and here we investigate the feasibility of concurrent CRT for LA‐NSCLC patients based on the PACIFIC criteria.

**Methods:**

We performed a retrospective study to evaluate the feasibility and efficacy of concurrent CRT prior to the approval of durvalumab. We assessed consecutive patients with LA‐NSCLC treated with CRT between January 2012 and June 2018.

**Results:**

We analyzed a total of 108 consecutive patients who received radical thoracic radiotherapy and concurrent platinum‐based chemotherapy. Of those patients, 105 (97%) completed the planned radiotherapy. Radiation pneumonitis was observed in 93 patients (85%), with a median of 130 days (range: 41–317 days) from the initiation of radiation to the onset of the complication. Among the patients, 74 (69%) were considered eligible for consolidation therapy with durvalumab. The overall response rate was 64%, and the two‐year survival rate was 63%. Patients who received an ICI after relapse were associated with significantly better survival than those who did not receive an ICI (two‐year survival rate: 87% vs. 41%, respectively; *P* = 0.001).

**Conclusions:**

Prior to the approval of durvalumab, the clinical application of ICIs improved the outcome of patients with relapsed NSCLC after CRT for LA‐NSCLC. The management of radiation pneumonitis remains a challenge following the approval of durvalumab.

## Introduction

The current standard treatment for unresectable locally advanced (LA) non‐small cell lung cancer (NSCLC) is definitive concurrent chemoradiotherapy (CRT). The efficacy of thoracic radiotherapy (TRT) for LA‐NSCLC was evaluated in 1968 when 800 patients with LA‐NSCLC were treated with 40–50 Gy of TRT. The overall survival (OS) was significantly improved in the TRT group compared with the placebo group (4.6 vs. 3.7 months, respectively).^1^ In 1990, a combination of TRT with cisplatin plus vinblastine significantly prolonged survival compared with TRT alone (OS: 13.7 vs. 9.6 months, respectively; *P* = 0.0066),^2^ and CRT has been positioned as the standard of care for patients with LA‐NSCLC.^3^, ^4^, ^5^ In recent years, a clinical trial compared cisplatin plus pemetrexed with cisplatin plus etoposide for TRT 60–66 Gy as a combination chemotherapy regimen. However, the results did not show a significant improvement in OS (OS: 26.8 vs. 25.0 months, respectively; hazard ratio [HR]: 0.98; 95% confidence interval [CI]: 0.79–1.20; *P* = 0.831).^6^

In the past 20 years, there have been no improvements in outcome (two‐year survival rate: 40%–60%).^3^, ^6^, ^7^, ^8^ However, in the PACIFIC Trial, concurrent CRT followed by consolidation therapy with durvalumab resulted in a significant prolongation of progression‐free survival (PFS) compared with placebo (PFS: 17.2 vs. 5.6 months, respectively; stratified HR, 0.51; 95% CI: 0.41–0.63) and the OS rate at 24 months (66.3% vs. 55.6%, respectively; stratified HR: 0.68; 99.73% CI: 0.47–0.997).^9^, ^10^ Based on the results of this study, durvalumab was approved in Japan in July 2018 as consolidation therapy after CRT. The main inclusion criteria in the PACIFIC Trial were (i) patients with stage III, unresectable NSCLC; (ii) patients who had received two or more cycles of platinum‐based chemotherapy concurrently with TRT (54–66 Gy), in which the mean lung dose was <20 Gy, the V20 (the volume of lung parenchyma that received ≥20 Gy) was <35%, or both; (iii) absence of disease progression after CRT; (iv) age ≥ 18 years; (v) a World Health Organization performance status (PS) of 0–1; (vi) an estimated life expectancy ≥12 weeks; and (vii) completion of the last radiation dose within 1–42 days prior to randomization of consolidation therapy with durvalumab. Key exclusion criteria were active or previous autoimmune disease (within the previous two years) or a history of primary immunodeficiency; evidence of uncontrolled, concurrent illness, or ongoing or active infections; unresolved toxic effects of grade ≥ 2 (according to the Common Terminology Criteria for Adverse Events [CTCAE]); and grade ≥ 2 pneumonitis from previous CRT.^9^ It is thought that the proportion of patients meeting the criteria of the PACIFIC Trial who should receive consolidation therapy with durvalumab is limited in clinical practice. In addition, new challenges in the management of side effects, such as radiation pneumonitis, have arisen.

Checkpoint immunotherapy has demonstrated high efficacy in numerous types of cancer,^11^, ^12^ including NSCLC. Prior to the approval of durvalumab, nivolumab^13^, ^14^ (December 2015), pembrolizumab^15^ (December 2016), and atezolizumab^16^ (January 2018) were approved in Japan as the second or subsequent line of therapy against advanced or recurrent NSCLC. Moreover, pembrolizumab monotherapy^17^ became available as the initial chemotherapy for programmed death ligand‐1‐positive advanced NSCLC in December 2016. Furthermore, in December 2018, the use of pembrolizumab^18^, ^19^ or atezolizumab^20^ plus chemotherapy was expanded to the first‐line treatment of metastatic NSCLC. The use of immune checkpoint inhibitors (ICIs) showed durable clinical benefit and long‐term remission in some patients,^21^, ^22^, ^23^ and has altered the standard of care for patients with metastatic NSCLC.

Now that the clinical issues related to the use of ICI for LA‐NSCLC patients in clinical practice are expected, it is considered important to evaluate patients who are effective in CRT. In this study, we investigated the feasibility of concurrent CRT for LA‐NSCLC patients based on the PACIFIC criteria. Furthermore, while ICIs have been used widely for the treatment of advanced‐stage disease, we retrospectively assessed consecutive patients with LA‐NSCLC treated with concurrent CRT (prior to the approval of durvalumab) in clinical practice to investigate the feasibility of this regimen.

## Methods

### Study patients and collection of clinical data

This retrospective study included 108 consecutive patients with LA‐NSCLC treated with CRT at the Kitasato University Hospital (Kanagawa, Japan) between January 2012 and June 2018. All patients underwent computed tomography (CT) for three‐dimensional conformal TRT and received concurrent TRT in combination with platinum‐based chemotherapy. We examined the clinical records (e.g., date of diagnosis, sex, smoking status, tumor–node–metastasis staging, histology, mutational status, PS, course of treatment, adverse events [AEs] including radiation pneumonitis, and survival time) of each patient.

### Thoracic radiotherapy

All patients were treated using three‐dimensional conformal radiotherapy. A free breathing computed tomography was performed for the treatment planning. The gross tumor volume (GTV) encompassed the primary tumor and involved the lymph nodes. The clinical target volume (CTV) boost was defined as the GTV plus a 5–10 mm margin. The CTV boost merged with elective nodal areas created the CTV total. The planning target volume (PTV) boost was defined as the CTV boost plus a 5–10 mm margin. The PTV total was defined as the CTV total plus a 5–10 mm margin. The majority of patients were treated using anteroposterior/posteroanterior opposing fields of up to 40 Gy to PTV total. Additionally, patients were treated using the off‐cord oblique field of up to 20 Gy to PTV boost. A V20 (the percent volume of the normal lung receiving 20 Gy or greater) was calculated on a dose‐volume histogram and should not exceed 35%.

### Evaluation of response and survival

Tumor response was classified in accordance with the Response Evaluation Criteria for Solid Tumors (version 1.1), based on the results of a complete medical history, physical examination, chest X‐ray examination, CT of the chest and abdomen, and other procedures (i.e., magnetic resonance imaging of the brain, positron emission tomography‐CT, and bone scintigraphy). Survival was defined as the time from the initiation of TRT to the date of documentation of treatment failure (death or disease progression) or the date of censoring at the final follow‐up examination (data cutoff: 30 June 2018; follow‐up time: median 20.5 months; range: 0.9–79.5 months). Patients with LA‐NSCLC were classified according to the initiation of TRT from January 2012 to June 2015 (2012–2015 cohort) and from July 2015 to June 2018 (2015–2018 cohort) to compare the short‐term survival between two recent cohorts.

### Statistical analysis

All survival analyses were performed using the Kaplan‐Meier method. Survival rates between subgroups were compared using the log‐rank test. To assess the interaction between the groups, interaction terms were included in the Cox proportional hazards model. The interaction was considered significant if *P* < 0.10. All analyses were performed using the SPSS software program, version 25.0 (SPSS, IBM Corp., Armonk, NY, USA).

This study was approved by the Kitasato University Medical Ethics Organization (B18‐216), which waived the requirement for patient informed consent due to the retrospective nature of the study.

## Results

### Patient characteristics

In total, 108 patients were treated with concurrent CRT combined with platinum‐based chemotherapy. Of these, 52 patients were treated between January 2012 and June 2015 and 56 patients were treated between July 2015 and June 2018 (Fig S1a). The characteristics of the patients included in this study are shown in Table 1. The median age was 65 years (range: 36–76 years), 81 of the patients (75%) were male, and only 11 patients (10%) were non‐smokers. Notably, 46 patients (43%) had adenocarcinoma, including two patients with epidermal growth factor receptor (*EGFR*) mutations, four patients with anaplastic lymphoma kinase (ALK) rearrangements, and one patient with ROS1 mutation. In addition, 38 patients (35%) had squamous cell carcinoma.

**Table 1:** Characteristics of the patients in this study (*n* = 108)

	All		2012/1–2015/6		2015/7–2018/6	
	No.	%	No.	%	No.	%
Patients, *n*	108	(100)	52	(48)	56	(52)
Age, years, median (range)	65 (36–76)		64 (36–75)		66 (38–76)	
Sex						
Male	81	(75)	38	(73)	43	(77)
Female	27	(25)	14	(27)	13	(23)
Smoking status						
Never	11	(10)	9	(17)	2	(4)
Former	27	(25)	14	(27)	13	(23)
Current	70	(65)	29	(56)	41	(73)
ECOG performance status						
0	46	(43)	16	(31)	30	(54)
1	60	(56)	34	(65)	26	(46)
2	2	(2)	2	(4)	0	(0)
Histology						
Ad	46	(43)	18	(35)	28	(50)
Sq	38	(35)	18	(35)	20	(36)
NOS	22	(20)	15	(28)	7	(12)
AdSq	2	(2)	1	(2)	1	(2)
Clinical stage						
IIIA	44	(41)	17	(33)	27	(48)
IIIB	64	(59)	35	(67)	29	(52)
Mutational status						
Wild‐type	69	(62)	30	(57)	39	(70)
ALK	4	(4)	2	(4)	2	(3)
EGFR	2	(2)	2	(4)	0	(0)
ROS1	1	(1)	0	(0)	1	(2)
Unknown	32	(30)	18	(35)	14	(25)

Ad, adenocarcinoma; AdSq, adenosquamous carcinoma; ALK, anaplastic lymphoma kinase; ECOG, Eastern Cooperative Oncology Group; EGFR, epidermal growth factor receptor.; NOS, not otherwise specified; Sq, squamous cell carcinoma.

We confirmed that patients were eligible to receive consolidation therapy with durvalumab based on the criteria of the PACIFIC Trial.^9^ In our cohort, 74 patients (69%) who received concurrent CRT met these criteria (Fig S1b, Table S1). Reasons for ineligibility were AEs of grade ≥ 2 after CRT (*n* = 9), disease progression (*n* = 8), grade 2 radiation pneumonitis (*n* = 6), receiving only one cycle of chemotherapy (*n* = 5), interstitial pneumonia combined (*n* = 3), and other complications (e.g., rheumatoid arthritis, colon cancer, and angina) (*n* = 3).

### Chemoradiotherapy for patients with LA‐NSCLC in clinical practice

All patients received radical TRT using a three‐dimensional planning system with a median 60 Gy dose (range: 10–66 Gy) and concurrent platinum‐based chemotherapy. The chemotherapy regimens used most commonly were cisplatin plus vinorelbine in 83 patients (77%) and weekly carboplatin plus paclitaxel in 19 patients (18%). Notably, 79 patients (73%) received one or two cycles of consolidation chemotherapy with the same regimen. Of the 82 patients who relapsed after CRT, 43 patients (52%) received cytotoxic drugs, 18 patients (22%) received treatment with an ICI, and seven patients (9%) received targeted tyrosine kinase inhibitors (TKI) in the course of treatment. Treatment details are shown in Table 2.

**Table 2:** Treatment of patients with locally advanced non‐small cell lung cancer (NSCLC) (*n* = 108)

	All	
	No.	%
Duration of radiation, days, median (range)	43 (4–68)	
Dose of radiation, Gy, median (range)	60 (10–66)	
V20, %, median (range†)	22 (3–37)	
V5, %, median (range†)	33 (5–54)	
Mean lung dose, Gy, median (range†)	12 (2–19)	
Regimen of chemotherapy		
CDDP+VNR	83	(77)
wCBDCA+PTX	19	(17)
CDDP+DOC	3	(3)
CDDP+S‐1	3	(3)
Cycles of chemotherapy		
1 / 2	16 / 13	(15 / 12)
3 / 4	16 / 63	(15 / 58)
Chemotherapy after recurrence		
Cytotoxic / ICI / TKI	43 / 18 / 7	(40 / 17 / 6)
Radiation (brain) / OPE / BSC	30 (20) / 3 / 14	(28 (19) / 3 / 13)
Number of regimens after recurrence		
0 / 1 / 2 / ≥3	27 / 32 / 14 / 9	(25 / 30 / 13 / 8)

† Excluding four patients (two with disease progression, one with tracheoesophageal fistula, and one with atelectasis).

BSC, best supportive care; CDDP, cisplatin; DOC, docetaxel; ICI, immune checkpoint inhibitor; OPE, operation; PTX, paclitaxel; TKI, tyrosine kinase inhibitor; VNR, vinorelbine; wCBDCA, weekly carboplatin.

### Safety of chemoradiotherapy

A total of 105 patients (97%) completed the planned radiotherapy. The reasons for discontinuing radiotherapy were hemoptysis at 10 Gy, esophageal tracheal fistula at 32 Gy, and paralysis due to spinal cord invasion of the primary lesion at 50 Gy. Radiation pneumonitis was observed in 93 patients (85%), with a median of 130 days (range: 41–317 days) from the initiation of radiation to the onset of the complication. Among these, 63 patients (58%) developed grade 1 radiation pneumonitis, while 30 patients (28%) developed grade 2–3 radiation pneumonitis, including 27 patients (25%) who required the administration of steroids (Table 3). High levels of V20 (%), V5 (%), and mean lung dose (Gy) for TRT were related to grade 2–3 pneumonitis. However, the pretreatment level of KL‐6, a marker of interstitial pneumonia, was not associated with the severity of radiation pneumonitis (Fig. S2). Nine patients experienced nonhematological toxic effects of CTCAE grade ≥ 2 from previous CRT, which included anorexia (*n* = 4), febrile neutropenia (*n* = 1), numbness (*n* = 1), tracheoesophageal fistula (*n* = 1), pyothorax (*n* = 1), and sepsis (*n* = 1).

**Table 3:** Efficacy and safety of chemoradiotherapy in patients with locally advanced non‐small cell lung cancer (NSCLC) (*n* = 108)

	All	
	No.	%
Radical CRT		
Completed	105	(97)
Canceled	3	(3)
(AE / exacerbation)	[2 / 1]	(2 / 1)
Chemotherapy ≥ 2 cycles		
Completed	92	(85)
Canceled	16	(15)
(AE / exacerbation)	[15 / 1]	(14 / 1)
Response of CRT		
CR / PR / SD / PD / NE	1 / 68 / 18 / 8 / 13	(1 / 63 / 17 / 7 / 12)
Site of recurrence, *n* = 78†		
Primary	25	(23)
Brain	16	(15)
Lung / bone / liver	6 / 4 / 1	(6 / 4 / 1)
Lymph node / pleura / subcutaneous	3 / 1 / 1	(3 / 1 / 1)
Multiple	21	(19)
radiation pneumonitis		
Gr0 / Gr1 / Gr2 / Gr3 / NE (required steroid treatment)	12 / 63 / 28 / 1 / 4 27	(11 / 58 / 26 / 1 / 4) (25)

† Patients died without evaluation of recurrent sites (*n* = 4).

AE, adverse event; CR, complete response; CRT, chemoradiotherapy; Gr, grade; NE, not evaluated; PD, progressive disease; PR, partial response; SD, stable disease.

### Treatment outcomes in patients with LA‐NSCLC

**Figure 1:**
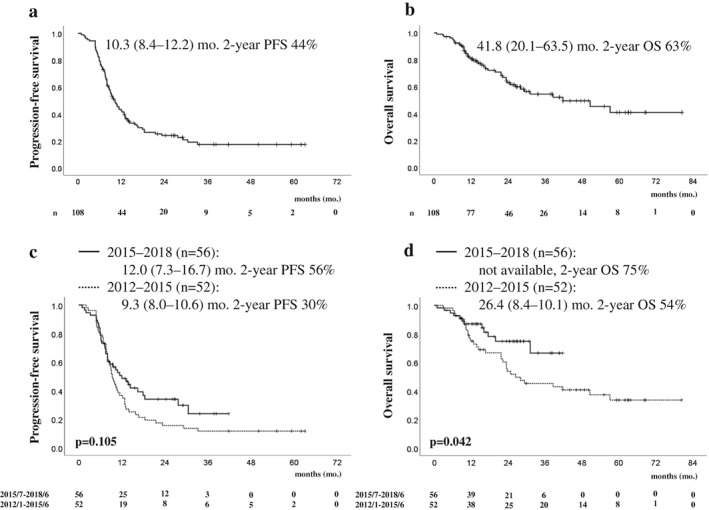
Kaplan‐Meier analysis‐based estimates of survival in patients with locally advanced non‐small cell lung cancer (LA‐NSCLC) treated with chemoradiotherapy based on the treatment periods (*n* = 108). **(a, b)** Progression‐free survival (PFS) and overall survival in all patients with LA‐NSCLC in this study. (**c, d)** Comparison of survival between patients treated from January 2012 to June 2015 (*n* = 52; broken line) and those treated from July 2015 to December 2018 (*n* = 56; solid line). *P*‐values were determined using the log‐rank test. Survival times in each group are indicated as medians (95% confidence interval) in months and the two‐year survival rate (%).

**Figure 2:**
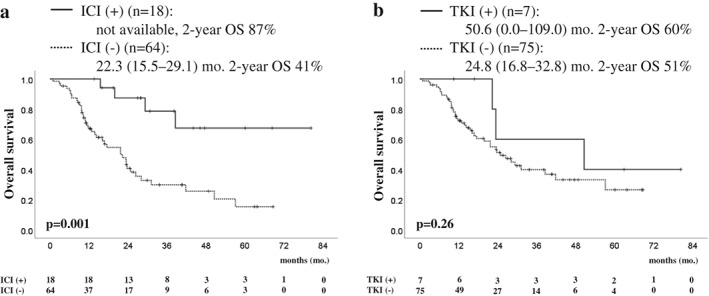
Comparison of overall survival in relapsed patients after chemoradiotherapy for locally advanced disease (*n* = 82). (**a**) Kaplan‐Meier analysis‐based estimates of survival of patients treated with an immune checkpoint inhibitor (ICI) (solid line) and those treated without an ICI (broken line). (**b**) Kaplan‐Meier analysis‐based estimates of survival of patients treated with tyrosine kinase inhibitors (TKIs) targeted to EGFR, ALK, and ROS1 (solid line) and those treated without TKIs (broken line). *P*‐values were determined using the log‐rank test. Survival times in each group are indicated as medians (95% confidence interval) in months and the two‐year survival rate (%). EGFR, epidermal growth factor receptor; ALK, anaplastic lymphoma kinase.

The overall response rate to CRT was 64% (Table 3) and the PFS was 10.3 months (95% CI: 8.4–12.2) (Fig. 1a). The OS was 41.8 months (95% CI: 20.1–63.5), with two‐ and five‐year survival rates of 63% and 41%, respectively (Fig. 1b). Although the PFS was not significantly different between the two groups (Fig. 1c), the OS was significantly better in the 2015–2018 cohort than in the 2012–2015 cohort, with a two‐year survival rate of 75% vs. 54%, respectively (*P* = 0.042) (Fig. 1d). During the period of this study, the TRT planning method employed in our hospital was not changed. Therefore, we analyzed subsequent chemotherapy for patients with recurrent disease after CRT to assess the difference in OS. Of the 82 patients who relapsed after CRT, 18 patients received treatment with an ICI (i.e., nivolumab [*n* = 14], pembrolizumab [*n* = 3], and atezolizumab [*n* = 1]) (Table 2). Among the LA‐NSCLC patients who relapsed after CRT, the OS of those treated with ICIs was superior to that of patients not treated with an ICI. However, it was not superior to that of patients who received targeted treatment with a TKI (Fig. 2, Table S2).

The univariate analyses of the clinical characteristics revealed that treatment period (2015–2018 vs. 2012–2015; *P* = 0.046), administration of ICIs after recurrence (received vs. not received; *P* = 0.003), the PACIFIC criteria (eligible vs. ineligible; *P* = 0.028), and radiation pneumonitis (grade 1–3 vs. grade 0; *P* = 0.037) were significantly associated with OS. The multivariate analyses, adjusted for these cofactors, revealed that treatment with an ICI after the patients relapsed after CRT (HR: 0.23; 95% CI: 0.07–0.57; *P* = 0.003) and treatment period (HR 0.47; 95% CI: 0.22–0.996) were favorable prognostic factors (Table 4).

**Table 4:** Survival analyses of patients with locally advanced non‐small cell lung cancer (NSCLC) treated with chemoradiotherapy (*n* = 108)

	Univariate			Multivariate		
	HR	95% CI	*P*‐value	HR	95% CI	*P‐*value
Age ≥65 versus <65 years	1.14	0.63–2.08	0.67			
Performance status 1–2 versus 0	1.49	0.79–2.82	0.22			
Clinical stage IIIB versus IIIA	1.10	0.59–2.05	0.76			
Histology Sq versus non‐Sq	1.36	0.73–2.53	0.33			
Driver mutations EGFR/ALK/ROS1 (+) versus (−)	0.52	0.12–2.15	0.36			
Regimen of chemotherapy CBDCA versus CDDP	0.98	0.43–2.15	0.36			
Cohort by treatment period 2015–2018 versus 2012–2015	0.50	0.25–0.99	0.046	0.47	0.22–0.996	0.049
ICIs after recurrence ICI (+) versus (−)	0.21	0.07–0.58	0.003	0.23	0.080–0.64	0.005
PACIFIC criteria Eligible versus ineligible	0.50	0.27–0.93	0.028	0.69	0.36–1.34	0.28
Radiation pneumonitis Grade 1–3 versus Grade 0	0.39	0.16–0.94	0.037	0.96	0.49–1.96	0.96

HR, hazard ratio; CI, confidence interval; Sq, squamous cell carcinoma; EGFR, epidermal growth factor receptor; ALK, anaplastic lymphoma kinase; CBDCA, carboplatin; CDDP, cisplatin; ICI, immune checkpoint inhibitor.

## Discussion

This retrospective study evaluated the outcome of concurrent CRT with platinum‐based chemotherapy. The results summarize the current status with regard to the efficacy and safety for the treatment of patients with LA‐NSCLC in clinical practice prior to the approval of durvalumab. Based on clinical trials involving patients with unresectable LA‐NSCLC, the treatment outcome has not improved for more than a decade, although several treatment strategies have been evaluated in clinical trials.^3^, ^4^, ^5^, ^6^, ^7^, ^24^, ^25^, ^26^, ^27^ The efficacy of consolidation therapy with cytotoxic drugs after CRT also failed to demonstrate an improved prognosis.^8^, ^28^ The PACIFIC Trial evaluated consolidation therapy with durvalumab after concurrent CRT for the treatment of patients with unresectable LA‐NSCLC, yielding clinically meaningful results.^9^, ^10^ The results of this trial revealed that durvalumab significantly improved PFS versus placebo (17.2 vs. 5.6 months, respectively; HR: 0.51; 95% CI: 0.41–0.63) and OS (stratified HR: 0.68; 99.73% CI: 0.47–0.997; *P* = 0.0025). The two‐year survival rates in the durvalumab and placebo groups were 66.3% (95% CI: 61.7–70.4) and 55.6% (95% CI: 48.9–61.8), respectively (*P* = 0.005).^10^ These findings indicated that durvalumab is an effective treatment option for patients with LA‐NSCLC who meet the PACIFIC criteria. However, it is necessary to accumulate additional data regarding the identification of patients who can receive consolidation therapy with durvalumab in the clinical setting. In our retrospective study, concurrent CRT for the treatment of unresectable LA‐NSCLC was effective in clinical practice, with acceptable toxicity, prior to the approval of durvalumab. The median OS was 41.8 months (95% CI: 20.1–65.5), two‐year survival was 63%, and five‐year survival was 41%.

Prior to their use in the treatment of LA‐NSCLC, ICIs were approved for metastatic or advanced NSCLC,^13^, ^14^, ^15^, ^16^, ^17^, ^18^, ^19^, ^20^ and have been widely used in daily practice owing to their safety and effectiveness. In previously treated patients with advanced NSCLC, the three‐year survival rate with nivolumab versus docetaxel was 17% (95% CI: 14–21) versus 8% (95% CI: 6–11), respectively.^23^ In a pooled population of relapsed patients with NSCLC (CHECKMATE 017 and 057), the four‐year updated survival rate with nivolumab was 14% (95% CI: 11–17).^29^ In a phase I study of pembrolizumab (KEYNOTE‐001), the three‐year survival rate was 26.4% (95% CI: 14.3–40.1) for treatment‐naive patients and 19.0% (95% CI: 15.0–23.4) for previously treated patients.^30^ In a subgroup analysis of the KEYNOTE‐001 Trial, the efficacy of ICIs after radiotherapy was investigated in 38 of 97 patients with metastatic NSCLC who received extracranial radiotherapy prior to pembrolizumab. The results showed that treatment with an ICI was associated with significantly longer PFS (median: 6.3 [95% CI: 2.1–10.4] vs. 2.0 months [95% CI: 1.8–2.1], respectively; HR: 0.50 [95% CI: 0.30–0.84]) and OS (median: 11.6 [95% CI: 6.5–20.5] vs. 5.3 months [95% CI: 3.0–8.5], respectively; HR: 0.59 [95% CI: 0.36–0.96]) compared with those reported in patients who did not receive prior extracranial radiotherapy.^31^ In this study, patients with LA‐NSCLC who relapsed after CRT and were subsequently treated with ICIs were associated with better prognosis than those who did not receive treatment with ICIs (two‐year survival rate: 87% vs. 41%, respectively; *P* = 0.001). Reanalysis of survival from recurrence after CRT to death also showed that patients who used ICI had a significantly better survival compared to those who did not (HR: 0.19 [95% CI: 0.068–0.55], *P* = 0.002). There is an increasing body of preclinical and clinical data regarding the radiotherapy‐induced immunomodulatory effects in the local tumor microenvironment, supporting the combination strategy.^32^, ^33^, ^34^ Radiotherapy modifies the tumor microenvironment, including enhanced antigen presentation, and the upregulation of tumor programmed death ligand‐1 and major histocompatibility complex class I expression.^32^, ^35^, ^36^, ^37^, ^38^ In addition to the local effects of irradiation at the tumor site, radiotherapy can also mediate an abscopal effect, which is linked to an immune‐mediated mechanism.^39^, ^40^, ^41^ The combination of radiotherapy and immunotherapy is considered an effective treatment strategy. In our study, the use of ICI for recurrence after CRT was a favorite prognostic factor for the patients with LA‐NSCLC.

In the PACIFIC Trial, the eligibility criteria were stringent compared with those in routine clinical practice. Thus, 74 of the 108 patients (69%) who received concurrent CRT with platinum‐based chemotherapy in our study met the PACIFIC criteria in the clinical setting. Regarding the schedule of the PACIFIC trial, the patients with LA‐NSCLC were randomized for consolidation therapy with durvalumab within 42 days after the completion of TRT based on the concept of immunomodulation through radiotherapy. Furthermore, in the subgroup analysis, the therapeutic effects of early initiation (i.e., within 14 days) were high, and the early use of an ICI was expected. On the other hand, even in relapsed patients with advanced or metastatic NSCLC, previous radiotherapy may increase the effect of treatment with an ICI,^31^ although the mechanism involved in this process is currently unclear. The optimal treatment method, namely the timing and duration of immunotherapy combined with radiotherapy, should be verified. Moreover, appropriate patient selection should be performed using reliable and predictive biomarkers, cytotoxic T cell infiltration and functionality, status of major histocompatibility complex expression, neoantigen burden, metabolic status, and general immune status factors (eg, neutrophil‐to‐lymphocyte ratio).^42^, ^43^

Radiation pneumonitis occurs frequently after TRT,^44^ and is characterized by clinically significant toxicity. In our study, radiation pneumonitis was found in approximately 90% of patients. However, its severity was mild/moderate in the majority of cases (CTCAE grade 1: 58%; grade 2: 26%; and grade 3: 1%), and the complication was manageable according to the appropriate radiotherapy plan. In the PACIFIC Trial, the incidence of AEs of any cause was similar in the durvalumab group versus the placebo group for AEs of any grade (97% vs. 95%, respectively), grade 3–4 AEs (30% vs. 26%, respectively), and serious AEs (29% vs. 23%, respectively).^9^ The most common grade 3–4 AEs were pneumonia (4% vs. 4%, respectively) and pneumonitis (3% vs. 3%, respectively). In several retrospective analyses, patients with advanced NSCLC who had received TRT and nivolumab exhibited a higher incidence of drug‐induced interstitial lung disease than those without TRT.^45^, ^46^ In the future, real‐world data will also be important in selecting the appropriate patients and establishing optimal treatment strategies (e.g., radiation dose, fractionation, field volume, schedule of treatment with ICIs, and treatment period) to combine radiotherapy with ICIs. Furthermore, elucidation of the effects of immune activation by ICIs on radiation pneumonitis is also required.

This study had certain limitations. First, this was a retrospective study conducted at a single institution with a small sample size; hence, the results cannot be considered definitive. Second, the current study included patients with LA‐SCLC who could receive TRT concurrent with platinum‐based chemotherapy; thus, it does not represent the overall status of stage III LA‐NSCLC in clinical practice. Third, several biases such as patient selection and treatment course should be considered between patients who could receive ICIs after relapse and those who could not. ICIs are considered an important treatment option for patients with LA‐NSCLC in whom TRT is indicated. Nevertheless, this study did not provide clear data regarding the treatment methods, such as timing and duration of use.

In conclusion, this retrospective study demonstrated the prognostic improvement in patients with LA‐NSCLC treated with concurrent CRT, followed by treatment with ICIs in the clinical setting even before the approval of durvalumab. The occurrence of radiation pneumonitis and drug‐induced interstitial lung disease poses a challenge to this treatment strategy. Therefore, appropriate patient selection, based on clinical factors and predictive markers, should be considered for the development of optimal treatment strategies.

## Disclosure

The authors state that they have no conflicts of interest to declare.

## Supporting information


**Figure S1** Patients with locally advanced non‐small cell lung cancer (LA‐NSCLC) treated with chemoradiotherapy in this study (*n* = 108). (**a**) Distribution of patients with LA‐NSCLC based on the treatment periods. (**b**) Pie chart showing the rates (%) of eligible (black) and ineligible (gray) patients for durvalumab consolidation in clinical practice based on the criteria of the PACIFIC Trial.
**Figure S2** Development of radiation pneumonitis after chemoradiotherapy. Box plots showing the association between the grade of radiation pneumonitis and (a) V20 (%), (b) V5 (%), (c) mean lung dose (Gy) for thoracic radiation, or (d) levels of KL‐6 in the serum. *P*‐values were determined using the Mann‐Whitney U test.

**Table S1** Characteristics of all patients based on the criteria of the PACIFIC Trial (*n* = 108).
**Table S2** Characteristics of the relapsed patients after CRT based on subsequent treatment with or without an immune checkpoint inhibitor (ICI) (*n* = 82).

## References

[1] Roswit B , Patno ME , Rapp R *et al* The survival of patients with inoperable lung cancer: A large‐scale randomized study of radiation therapy versus placebo. Radiology 1968; 90 (4): 688–97. 4170866 10.1148/90.4.688

[2] Dillman RO , Seagren SL , Propert KJ *et al* A randomized trial of induction chemotherapy plus high‐dose radiation versus radiation alone in stage III non‐small‐cell lung cancer. N Engl J Med 1990; 323 (14): 940–5. 2169587 10.1056/NEJM199010043231403

[3] Auperin A , Le Pechoux C , Rolland E *et al* Meta‐analysis of concomitant versus sequential radiochemotherapy in locally advanced non‐small‐cell lung cancer. J Clin Oncol 2010; 28 (13): 2181–90. 20351327 10.1200/JCO.2009.26.2543

[4] Yamamoto N , Nakagawa K , Nishimura Y *et al* Phase III study comparing second‐ and third‐generation regimens with concurrent thoracic radiotherapy in patients with unresectable stage III non‐small‐cell lung cancer: West Japan thoracic oncology group WJTOG0105. J Clin Oncol 2010; 28 (23): 3739–45. 20625120 10.1200/JCO.2009.24.5050

[5] Segawa Y , Kiura K , Takigawa N *et al* Phase III trial comparing docetaxel and cisplatin combination chemotherapy with mitomycin, vindesine, and cisplatin combination chemotherapy with concurrent thoracic radiotherapy in locally advanced non‐small‐cell lung cancer: OLCSG 0007. J Clin Oncol 2010; 28 (20): 3299–306. 20530281 10.1200/JCO.2009.24.7577

[6] Senan S , Brade A , Wang LH *et al* PROCLAIM: Randomized phase III trial of Pemetrexed‐cisplatin or etoposide‐cisplatin plus thoracic radiation therapy followed by consolidation chemotherapy in locally advanced nonsquamous non‐small‐cell lung cancer. J Clin Oncol 2016; 34 (9): 953–62. 26811519 10.1200/JCO.2015.64.8824

[7] Furuse K , Fukuoka M , Kawahara M *et al* Phase III study of concurrent versus sequential thoracic radiotherapy in combination with mitomycin, vindesine, and cisplatin in unresectable stage III non‐small‐cell lung cancer. J Clin Oncol 1999; 17 (9): 2692–9. 10561343 10.1200/JCO.1999.17.9.2692

[8] Tsujino K , Kurata T , Yamamoto S *et al* Is consolidation chemotherapy after concurrent chemo‐radiotherapy beneficial for patients with locally advanced non‐small‐cell lung cancer? A pooled analysis of the literature. J Thorac Oncol 2013; 8 (9): 1181–9. 23883782 10.1097/JTO.0b013e3182988348

[9] Antonia SJ , Villegas A , Daniel D *et al* Durvalumab after chemoradiotherapy in stage III non‐small‐cell lung cancer. N Engl J Med 2017; 377 (20): 1919–29. 28885881 10.1056/NEJMoa1709937

[10] Antonia SJ , Villegas A , Daniel D *et al* Overall survival with durvalumab after chemoradiotherapy in stage III NSCLC. N Engl J Med 2018; 379: 342–235. 10.1056/NEJMoa1809697 30280658

[11] Wang X , Bao Z , Zhang X *et al* Effectiveness and safety of PD‐1/PD‐L1 inhibitors in the treatment of solid tumors: A systematic review and meta‐analysis. Oncotarget 2017; 8 (35): 59901–14. 28938692 10.18632/oncotarget.18316 PMC5601788

[12] Salama AK , Moschos SJ . Next steps in immuno‐oncology: Enhancing antitumor effects through appropriate patient selection and rationally designed combination strategies. Ann Oncol 2017; 28 (1): 57–74. 28177433 10.1093/annonc/mdw534 PMC6887913

[13] Brahmer J , Reckamp KL , Baas P *et al* Nivolumab versus docetaxel in advanced squamous‐cell non‐small‐cell lung cancer. N Engl J Med 2015; 373 (2): 123–35. 26028407 10.1056/NEJMoa1504627 PMC4681400

[14] Borghaei H , Paz‐Ares L , Horn L *et al* Nivolumab versus docetaxel in advanced nonsquamous non‐small‐cell lung cancer. N Engl J Med 2015; 373 (17): 1627–39. 26412456 10.1056/NEJMoa1507643 PMC5705936

[15] Herbst RS , Baas P , Kim DW *et al* Pembrolizumab versus docetaxel for previously treated, PD‐L1‐positive, advanced non‐small‐cell lung cancer (KEYNOTE‐010): A randomised controlled trial. Lancet 2016; 387 (10027): 1540–50. 26712084 10.1016/S0140-6736(15)01281-7

[16] Rittmeyer A , Barlesi F , Waterkamp D *et al* Atezolizumab versus docetaxel in patients with previously treated non‐small‐cell lung cancer (OAK): A phase 3, open‐label, multicentre randomised controlled trial. Lancet 2017; 389 (10066): 255–65. 27979383 10.1016/S0140-6736(16)32517-X PMC6886121

[17] Reck M , Rodriguez‐Abreu D , Robinson AG *et al* Pembrolizumab versus chemotherapy for PD‐L1‐positive non‐small‐cell lung cancer. N Engl J Med 2016; 375 (19): 1823–33. 27718847 10.1056/NEJMoa1606774

[18] Gandhi L , Rodriguez‐Abreu D , Gadgeel S *et al* Pembrolizumab plus chemotherapy in metastatic non‐small‐cell lung cancer. N Engl J Med 2018; 378 (22): 2078–92. 29658856 10.1056/NEJMoa1801005

[19] Paz‐Ares L , Luft A , Vicente D *et al* Pembrolizumab plus chemotherapy for squamous non‐small‐cell lung cancer. N Engl J Med 2018; 379 (21): 2040–51. 30280635 10.1056/NEJMoa1810865

[20] Socinski MA , Jotte RM , Cappuzzo F *et al* Atezolizumab for first‐line treatment of metastatic nonsquamous NSCLC. N Engl J Med 2018; 378 (24): 2288–301. 29863955 10.1056/NEJMoa1716948

[21] Horn L , Spigel DR , Vokes EE *et al* Nivolumab versus docetaxel in previously treated patients with advanced non‐small‐cell lung cancer: Two‐year outcomes from two randomized, open‐label, phase III trials (CheckMate 017 and CheckMate 057). J Clin Oncol 2017; 35 (35): 3924–33. 29023213 10.1200/JCO.2017.74.3062 PMC6075826

[22] Gettinger S , Horn L , Jackman D *et al* Five‐year follow‐up of Nivolumab in previously treated advanced non‐small‐cell lung cancer: Results from the CA209‐003 study. J Clin Oncol 2018; 36 (17): 1675–84. 29570421 10.1200/JCO.2017.77.0412

[23] Vokes EE , Ready N , Felip E *et al* Nivolumab versus docetaxel in previously treated advanced non‐small‐cell lung cancer (CheckMate 017 and CheckMate 057): 3‐year update and outcomes in patients with liver metastases. Ann Oncol 2018; 29 (4): 959–65. 29408986 10.1093/annonc/mdy041

[24] Cheema PK , Rothenstein J , Melosky B , Brade A , Hirsh V . Perspectives on treatment advances for stage III locally advanced unresectable non‐small‐cell lung cancer. Curr Oncol 2019; 26 (1): 37–42. 30853796 10.3747/co.25.4096 PMC6380636

[25] Bradley JD , Paulus R , Komaki R *et al* Standard‐dose versus high‐dose conformal radiotherapy with concurrent and consolidation carboplatin plus paclitaxel with or without cetuximab for patients with stage IIIA or IIIB non‐small‐cell lung cancer (RTOG 0617): A randomised, two‐by‐two factorial phase 3 study. Lancet Oncol 2015; 16 (2): 187–99. 25601342 10.1016/S1470-2045(14)71207-0 PMC4419359

[26] Oh IJ , Kim KS , Kim YC *et al* A phase III concurrent chemoradiotherapy trial with cisplatin and paclitaxel or docetaxel or gemcitabine in unresectable non‐small cell lung cancer: KASLC 0401. Cancer Chemother Pharmacol 2013; 72 (6): 1247–54. 24091849 10.1007/s00280-013-2308-5

[27] Liang J , Bi N , Wu S *et al* Etoposide and cisplatin versus paclitaxel and carboplatin with concurrent thoracic radiotherapy in unresectable stage III non‐small cell lung cancer: A multicenter randomized phase III trial. Ann Oncol 2017; 28 (4): 777–83. 28137739 10.1093/annonc/mdx009

[28] Hanna N , Neubauer M , Yiannoutsos C *et al* Phase III study of cisplatin, etoposide, and concurrent chest radiation with or without consolidation docetaxel in patients with inoperable stage III non‐small‐cell lung cancer: The Hoosier oncology group and U.S. oncology. J Clin Oncol 2008; 26 (35): 5755–60. 19001323 10.1200/JCO.2008.17.7840

[29] Antonia SJ , Borghaei H , Ramalingam SS *et al* Four‐year survival with nivolumab in patients with previously treated advanced non‐small‐cell lung cancer: A pooled analysis. Lancet Oncol 2019; 20: 1395–408. 31422028 10.1016/S1470-2045(19)30407-3 PMC7193685

[30] Leighl NB , Hellmann MD , Hui R *et al* Pembrolizumab in patients with advanced non‐small‐cell lung cancer (KEYNOTE‐001): 3‐year results from an open‐label, phase 1 study. Lancet Respir Med 2019; 7 (4): 347–57. 30876831 10.1016/S2213-2600(18)30500-9

[31] Shaverdian N , Lisberg AE , Bornazyan K *et al* Previous radiotherapy and the clinical activity and toxicity of pembrolizumab in the treatment of non‐small‐cell lung cancer: A secondary analysis of the KEYNOTE‐001 phase 1 trial. Lancet Oncol 2017; 18 (7): 895–903. 28551359 10.1016/S1470-2045(17)30380-7 PMC5538772

[32] Ko EC , Raben D , Formenti SC . The integration of radiotherapy with immunotherapy for the treatment of non‐small cell lung cancer. Clin Cancer Res 2018; 24 (23): 5792–806. 29945993 10.1158/1078-0432.CCR-17-3620

[33] Kordbacheh T , Honeychurch J , Blackhall F , Faivre‐Finn C , Illidge T . Radiotherapy and anti‐PD‐1/PD‐L1 combinations in lung cancer: Building better translational research platforms. Ann Oncol 2018; 29 (2): 301–10. 29309540 10.1093/annonc/mdx790

[34] Filippi AR , Di Muzio J , Badellino S , Mantovani C , Ricardi U . Locally‐advanced non‐small cell lung cancer: Shall immunotherapy be a new chance? J Thorac Dis 2018; 10 (Suppl 13): S1461–7. 29951297 10.21037/jtd.2017.12.53 PMC5994493

[35] Deng L , Liang H , Burnette B *et al* Irradiation and anti‐PD‐L1 treatment synergistically promote antitumor immunity in mice. J Clin Invest 2014; 124 (2): 687–95. 24382348 10.1172/JCI67313 PMC3904601

[36] Dovedi SJ , Cheadle EJ , Popple AL *et al* Fractionated radiation therapy stimulates antitumor immunity mediated by both resident and infiltrating polyclonal T‐cell populations when combined with PD‐1 blockade. Clin Cancer Res 2017; 23 (18): 5514–26. 28533222 10.1158/1078-0432.CCR-16-1673

[37] Yoneda K , Kuwata T , Kanayama M *et al* Alteration in tumoural PD‐L1 expression and stromal CD8‐positive tumour‐infiltrating lymphocytes after concurrent chemo‐radiotherapy for non‐small cell lung cancer. Br J Cancer 2019; 121: 490–6. 31388183 10.1038/s41416-019-0541-3 PMC6738061

[38] Daly ME , Monjazeb AM , Kelly K . Clinical trials integrating immunotherapy and radiation for non‐small‐cell lung cancer. J Thorac Oncol 2015; 10 (12): 1685–93. 26484629 10.1097/JTO.0000000000000686

[39] Demaria S , Ng B , Devitt ML *et al* Ionizing radiation inhibition of distant untreated tumors (abscopal effect) is immune mediated. Int J Radiat Oncol Biol Phys 2004; 58 (3): 862–70. 14967443 10.1016/j.ijrobp.2003.09.012

[40] Grass GD , Krishna N , Kim S . The immune mechanisms of abscopal effect in radiation therapy. Curr Probl Cancer 2016; 40 (1): 10–24. 26612692 10.1016/j.currproblcancer.2015.10.003

[41] Ng J , Dai T . Radiation therapy and the abscopal effect: A concept comes of age. Ann Transl Med 2016; 4 (6): 118. 27127771 10.21037/atm.2016.01.32 PMC4828732

[42] Blank CU , Haanen JB , Ribas A , Schumacher TN . CANCER IMMUNOLOGY. The "cancer immunogram". Science 2016; 352 (6286): 658–60. 27151852 10.1126/science.aaf2834

[43] Fukui T , Okuma Y , Nakahara Y *et al* Activity of Nivolumab and utility of neutrophil‐to‐lymphocyte ratio as a predictive biomarker for advanced non‐small‐cell lung cancer: A prospective observational study. Clin Lung Cancer 2019; 20 (3): 208–214.e202. 29803573 10.1016/j.cllc.2018.04.021

[44] Simone CB 2nd . Thoracic radiation Normal tissue injury. Semin Radiat Oncol 2017; 27 (4): 370–7. 28865520 10.1016/j.semradonc.2017.04.009

[45] Kataoka Y , Ebi N , Fujimoto D *et al* Prior radiotherapy does not predict nivolumab response in non‐small‐cell lung cancer: A retrospective cohort study. Ann Oncol 2017; 28 (6): 1402. 28368440 10.1093/annonc/mdx114

[46] Tamiya A , Tamiya M , Nakahama K *et al* Correlation of radiation pneumonitis history before Nivolumab with onset of interstitial lung disease and progression‐free survival of patients with pre‐treated advanced non‐small cell lung cancer. Anticancer Res 2017; 37 (9): 5199–205. 28870955 10.21873/anticanres.11943

