# Supplementary material for: Current status and progress of concurrent chemoradiotherapy in patients with locally advanced non‐small cell lung cancer prior to the approval of durvalumab
Source: Thorac Cancer. 2020 Feb 14;11(4):1005–14. doi: 10.1111/1759-7714.13357 (PMC7113036; doi:10.1111/1759-7714.13357)
Supplement: Supplementary file 1 — Figure S1 Patients with locally advanced non‐small cell lung cancer (LA‐NSCLC) treated with chemoradiotherapy in this study (n = 108). (a) Distribution of patients with LA‐NSCLC based on the treatment periods. (b) Pie chart showing the rates (%) of eligible (black) and ineligible (gray) patients for durvalumab consolidation in clinical practice based on the criteria of the PACIFIC Trial. Figure S2 Development of radiation pneumonitis after chemoradiotherapy. Box plots showing the association between the grade of radiation pneumonitis and (a) V20 (%), (b) V5 (%), (c) mean lung dose (Gy) for thoracic radiation, or (d) levels of KL‐6 in the serum. P‐values were determined using the Mann‐Whitney U test. [file TCA-11-1005-s001.pptx]

## Slide 1
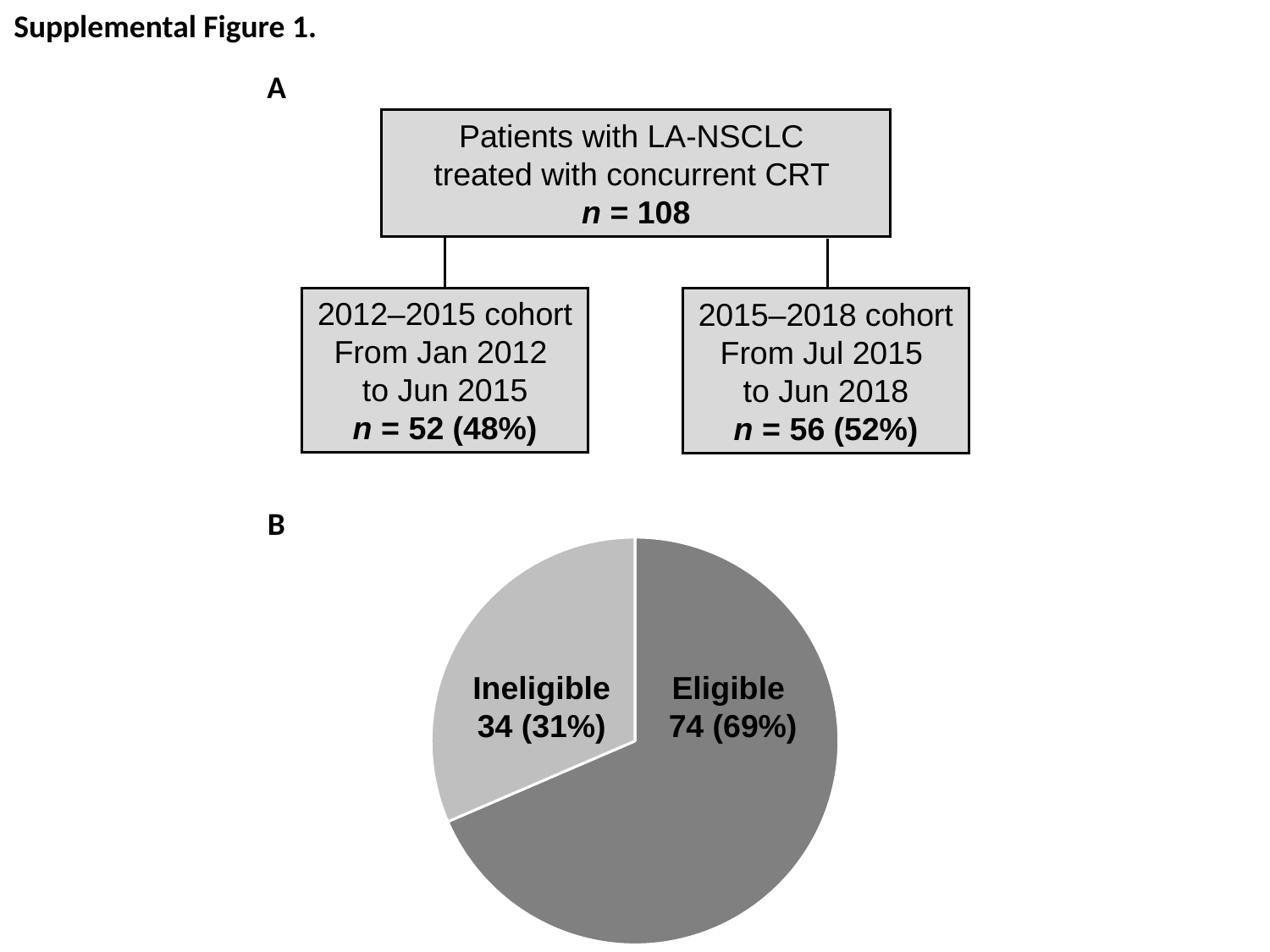

Supplemental Figure 1.
A
Patients with LA-NSCLC
treated with concurrent CRT
n = 108
2012–2015 cohort
From Jan 2012
to Jun 2015
n = 52 (48%)
2015–2018 cohort
From Jul 2015
to Jun 2018
n = 56 (52%)
B
### Chart
| Category | |
|---|---|
| PACIFIC内 | 68.51851851851852 |
| PACIFIC外 | 31.48148148148148 |Eligible
74 (69%)
Ineligible
34 (31%)

## Slide 2
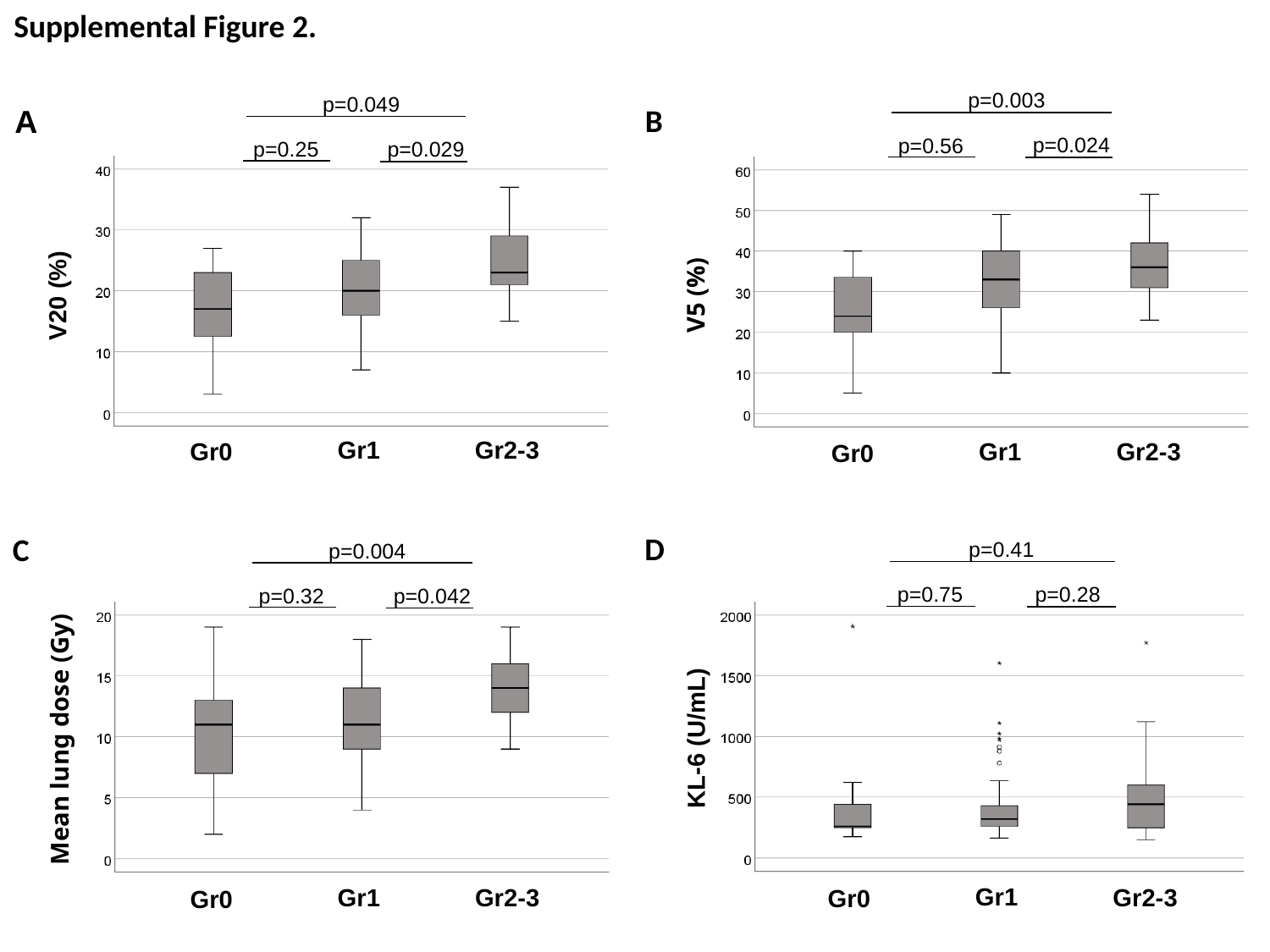

Supplemental Figure 2.
p=0.003
p=0.024
p=0.56
V5 (%)
V5 (%)
p=0.049
A
p=0.029
p=0.25
V20 (%)
B
Gr1
Gr2-3
Gr0
Gr1
Gr2-3
Gr0
D
C
p=0.41
p=0.28
p=0.75
KL-6 (U/mL)
p=0.004
p=0.042
p=0.32
Mean lung dose (Gy)
Gr1
Gr2-3
Gr1
Gr2-3
Gr0
Gr0
