# Supplementary material for: Current status and progress of concurrent chemoradiotherapy in patients with locally advanced non‐small cell lung cancer prior to the approval of durvalumab
Source: Thorac Cancer. 2020 Feb 14;11(4):1005–14. doi: 10.1111/1759-7714.13357 (PMC7113036; doi:10.1111/1759-7714.13357)
Supplement: Supplementary file 2 — Table S1 Characteristics of all patients based on the criteria of the PACIFIC Trial (n = 108). Table S2 Characteristics of the relapsed patients after CRT based on subsequent treatment with or without an immune checkpoint inhibitor (ICI) (n = 82). [file TCA-11-1005-s002.docx]

**Supplemental Table 1.** Characteristics of all patients based on the criteria of the PACIFIC Trial (*n* = 108)

|  | All | | PACIFIC eligible | | | PACIFIC ineligible | |
| --- | --- | --- | --- | --- | --- | --- | --- |
|  | No. | % | No. | % | No. | | % |
| Patients, *n* | 108 | (100) | 74 | (69) | 34 | | (31) |
| Age, years, median (range) | 65 (36–76) | | 63 (36–76) | | 68 (49–76) | | |
| Sex  Male  　Female | 81  27 | (75)  (25) | 54  20 | (73)  (27) | 26  7 | | (76)  (24) |
| Smoking status  Never  Former  Current | 11  27  70 | (10)  (25)  (65) | 9  15  50 | (12)  (20)  (68) | 2  12  20 | | (6)  (35)  (59) |
| ECOG performance status  0  1  2 | 46  60  2 | (43)  (56)  (2) | 35  38  1 | (31)  (65)  (4) | 11  22  1 | | (32)  (65)  (3) |
| Histology  Ad  Sq  NOS  AdSq | 46  38  22  2 | (43)  (35)  (20)  (2) | 33  27  12  2 | (45)  (36)  (16)  (3) | 13  11  10  0 | | (38)  (32)  (30)  (0) |
| Clinical stage  IIIA  IIIB | 44  64 | (41)  (59) | 32  42 | (43)  (57) | 12  22 | | (35)  (65) |
| Mutational status  Wild type  ALK  EGFR  ROS1  Unknown | 69  4  2  1  32 | (62)  (4)  (2)  (1)  (30) | 46  4  2  1  21 | (62)  (6)  (3)  (1)  (28) | 23  0  0  0  11 | | (68)  (0)  (0)  (0)  (32) |

ECOG, Eastern Cooperative Oncology Group; Ad, adenocarcinoma; Sq, squamous cell carcinoma; NOS, not otherwise specified; AdSq, adenosquamous carcinoma; ALK, anaplastic lymphoma kinase; EGFR, epidermal growth factor receptor.

**Supplemental Table 2.** Characteristics of the relapsed patients after CRT based on subsequent treatment with or without an immune checkpoint inhibitor (ICI) (*n* = 82)

|  | Relapsed patients | | With ICI | | | Without ICI | |
| --- | --- | --- | --- | --- | --- | --- | --- |
|  | No. | % | No. | % | No. | | % |
| Patients, *n* | 82 | (100) | 18 | (22) | 64 | | (78) |
| Age, years, median (range) | 64 (36–76) | | 63 (38–74) | | | 65 (36–76) | |
| Sex  Male  　Female | 59  23 | (72)  (28) | 11  7 | (61)  (39) | 48  16 | | (75)  (25) |
| Smoking status  Never  Former  Current | 10  18  54 | (12)  (22)  (66) | 1  6  11 | (6)  (33)  (61) | 9  12  43 | | (14)  (19)  (67) |
| ECOG performance status  0  1  2 | 30  50  2 | (37)  (61)  (2) | 7  11  0 | (39)  (61)  (0) | 23  39  2 | | (36)  (61)  (3) |
| Histology  Ad  Sq  NOS  AdSq | 32  30  18  2 | (39)  (37)  (22)  (2) | 8  9  1  0 | (44)  (50)  (6)  (0) | 24  21  17  2 | | (37)  (33)  (27)  (3) |
| Clinical stage  IIIA  IIIB | 30  52 | (37)  (63) | 4  14 | (22)  (78) | 26  38 | | (41)  (59) |
| Mutational status  Wild type  ALK  EGFR  ROS1  Unknown | 54  3  2  1  22 | (66)  (4)  (2)  (1)  (27) | 11  0  1  0  6 | (61)  (0)  (6)  (0)  (33) | 43  3  1  1  16 | | (67)  (4)  (2)  (2)  (25) |

CRT, chemoradiotherapy; ICI, immune checkpoint inhibitor; ECOG, Eastern Cooperative Oncology Group; Ad, adenocarcinoma; Sq, squamous cell carcinoma; NOS, not otherwise specified; AdSq, adenosquamous carcinoma; ALK, anaplastic lymphoma kinase; EGFR, epidermal growth factor receptor.
